# Media Use of Mothers, Media Use of Children, and Parent–Child Interaction Are Related to Behavioral Difficulties and Strengths of Children

**DOI:** 10.3390/ijerph16234651

**Published:** 2019-11-22

**Authors:** Tanja Poulain, Juliane Ludwig, Andreas Hiemisch, Anja Hilbert, Wieland Kiess

**Affiliations:** 1 LIFE Leipzig Research Center for Civilization Diseases, Leipzig University Medical Center, Philipp-Rosenthal-Strasse 27, 04103 Leipzig, Germany; jludwig@life.uni-leipzig.de (J.L.); wieland.kiess@medizin.uni-leipzig.de (W.K.); 2 Department of Women and Child Health, Hospital for Children and Adolescents and Center for Pediatric Research (CPL), Leipzig University Medical Center, Liebigstrasse 20a, 04103 Leipzig, Germany; andreas.hiemisch@medizin.uni-leipzig.de; 3 Integrated Research and Treatment Center AdiposityDiseases, Leipzig University Medical Center, Philipp-Rosenthal-Strasse 27, 04103 Leipzig, Germany; anja.hilbert@medizin.uni-leipzig.de; 4 Department of Medical Psychology and Medical Sociology and Department of Psychosomatic Medicine and Psychotherapy, Leipzig University Medical Center, Philipp-Rosenthal-Strasse 55, 04103 Leipzig, Germany

**Keywords:** media use, interaction, mother, children, behavioral difficulties, behavioral strengths

## Abstract

The present study investigated the associations of media use of children, media use of mothers, and parent-child interactions with behavioral strengths and difficulties in children. Screen time of 553 2- to 9-year-old children and their mothers were indicated by the daily durations of their TV/games console/computer/mobile phone use. The amount of parent–child interaction was indicated by the frequencies of shared activities at home. Behavioral strengths and difficulties of children were investigated using the Strengths and Difficulties Questionnaire. Children whose mothers reported high screen times (>/= 5 h/day) were significantly more likely to show high screen times (>/= 2 h/day). High screen time of children was associated with more conduct problems, more symptoms of hyperactivity/inattention and less prosocial behavior. High screen time of mothers was associated with emotional problems, conduct problems, and symptoms of hyperactivity/inattention. In contrast, a higher frequency of parent–child interactions was associated with fewer conduct problems, fewer peer-relationship problems, and more prosocial behavior of children. Children might use the media behavior of their mothers as a role model for their own media use. Furthermore, the findings suggest that media use of children and mothers and parent–child interaction contribute independently to behavioral strengths and difficulties of children.

## 1. Introduction

Television, tablets, computers, and smartphones have become technology devices that are common and accessible in nearly all families [1,2]. Furthermore, the use of electronic media, especially of interactive and mobile media, has increased substantially in the last years, not only in adolescents [3] but also in younger children [4]. According to a recent qualitative study, media multitasking, i.e., the exposure to or active use of several screen-based media in parallel, is common in young families, as are negotiations about media use [5]. Given the magnitude of studies indicating negative effects of high media use on social life, physical and mental health [6], cognitive skills, and brain functioning [7], this development is alarming.

In the context of behavioral strengths and difficulties of children, previous cross-sectional [8,9] as well as longitudinal [4,10,11] studies reported positive associations between screen time and behavioral difficulties, especially antisocial behavior and hyperactivity/inattention. Furthermore, excessive media use of children was shown to be associated with lower amounts of prosocial behavior [8,12]. Interestingly, this association is suggested to be partly mediated by the amount of parent-child interactions [8].

A new line of research investigates the link between media use of parents and behavioral difficulties of children. In cross-sectional studies, parent mobile technology use in the presence of the child has been shown to be associated with lower parental responsiveness, sensitivity, and attention [13,14], with more hostility [15], and, possibly as a consequence, with more risky and less attentive behavior in children [16,17]. Media use of mothers could also be linked to media use of children [18,19]. A recent study showed associations between maternal problematic use of electronic media and interruptions of parent–child interactions, which, in turn, were associated with more behavioral difficulties in 1- to 5-year-old children [20]. A longitudinal investigation indicated that associations between interrupted interactions and behavioral difficulties are bidirectional, at least for externalizing problems [21].

Similar to the media use of children and mothers, dysfunctional parent–child interactions have been shown to be associated with behavioral difficulties in young children [8,22]. Furthermore, infrequent interactions between parents and children could be linked to lower prosocial behavior in children [8], whereas positive parenting and parent–child attachment have been shown to be related to more social competence [23]. 

Overall, previous studies have suggested that screen time of children and parents and parent–child interaction are interrelated and associated with behavioral strengths and difficulties of children. However, to the best of our knowledge, no previous study investigated all three behaviors in the same study. The present study aimed to fill this research gap and, therefore, assessed (independent) associations of screen time of children, screen time of mothers, and the frequency of direct parent–child interactions with internalizing (emotional problems, peer-relationship problems) and externalizing (conduct problems, symptoms of hyperactivity/inattention) behavioral difficulties and prosocial behavior of children. Based on previous study findings [18,19], we expected the screen time of mothers to be positively related to the screen time of children, but to show negative associations with the frequency of parent–child interactions. Furthermore, screen time of children was expected to be associated with fewer amounts of parent–child interactions [8]. As could be shown in other studies [8,9,10,11,12,20,21], we hypothesized screen times of children and mothers to be related to more behavioral difficulties and less prosocial behavior. In contrast, parent–child interactions were expected to go along with fewer behavioral difficulties and more prosocial behavior [8,22].

## 2. Materials and Methods 

### 2.1. Subjects

The study was conducted within the framework of the LIFE Child study, a study investigating healthy child development from pregnancy to young adulthood [24,25]. Study participants are mainly recruited from the city and the surrounding areas of Leipzig. All children and parents who are interested in the study and are not suffering from any chronic, chromosomal, and syndromal diseases are invited to participate in the LIFE Child study. The study comprises several examinations and questionnaires, which were approved by the Ethics Committee of the Medical Faculty of the University of Leipzig (Reg. No. 264/10-ek).

For the present study, only cross-sectional data were analyzed. Data were collected between July 2017 and December 2018. The initial study sample included 773 children and their mothers. In the case of multiple children per family, only one child was chosen at random. In the case of multiple visits per child, only the last visit was considered. The final sample comprised 553 children aged between 2 and 9 years (55% boys, mean age = 6.21 years (SD = 2.31)). The mean age of mothers was 38.11 years (SD = 5.44). 

The socio-economic status (SES) of all study participants was assessed by a composite score that combines information on parental education, parental occupation, and the household equivalent income [26]. The score ranges between 3 and 21. Based on data collected in a representative German sample [26], cut-off scores were created that separate low (lowest 20%), middle (middle 60%), and high SES families (highest 20%). In the present sample, 5% of the participating families belonged to the low, 50% to the middle, and 45% to the high SES group, indicating a participant bias towards higher social strata.

### 2.2. Measurements

#### 2.2.1. Media Use

Media use was assessed using two versions of a parental media questionnaire, one on the media use of children, and another on the media use of mothers. The questions of both versions were highly comparable. The questionnaire was designed by the authors and represents an adaptation to a previous questionnaire which was based on the German Health Interview and Examination Survey for Children and Adolescents (KiGGS) [27] and was used in several research projects [4,28]. The adapted version investigates the same media types and uses the same response categories, but is more detailed (e.g., separates media use on weekdays and weekends and distinguishes between offline and online media use). It assesses how much time children (or mothers) usually spend using TV, games consoles, computers (including tablets, online and offline), and mobile phones (online and offline). Participants were asked to exclude media use in school or at work and media use without using the screen (e.g., for listening to music). For each question, participants had to choose one of five response categories ranging from “never” to “>4 h/day”. The responses were transformed into durations using the following algorithm: “never” = 0, “approximately 30 min/day” = 0.5, “1–2 h/day” = 1.5, “3–4 h/day = 3.5, “>4 h/day” = 5. The durations of week days and weekend were combined (final duration = (duration on week days × 5 + duration on weekend × 2)/7). Furthermore, offline and online computer (or mobile phone) use were summed up. Total screen time was assessed by summing up the durations of TV, games consoles, computer, and mobile phone use. 

#### 2.2.2. Parent–Child Interaction

The frequency of interactions between children and their parents was assessed using an adaptation to the German version of the questionnaire on preschool aged children’s activities in the family (Roßbach HG, Leal TB. Mütterfragebogen zu kindlichen Aktivitäten im Kontext des Familiensettings (AKFRA). Deutsche Fassung des Questionnnaire on Preschool-Aged Childrens’ Activities in the Family. Unpublished manuscript). The original questionnaire comprised 23–26 activities shared between young children and their parents at home. The adaptation contains only the 11 activities that are also relevant in older childhood (telling a story/reading, music, movement play, creative activities, playing with building blocks, puzzles, ball games, role play, language games, numbers games, and talking about problems). The questionnaire was completed by the participating mothers. For each item, they were asked to choose the most appropriate of the following six response categories: “never” (0), “once/month” (1), “every 2 weeks” (2), “once/week” (3), “more often than once/week” (4), “daily” (5). For further analysis, the sum score of all activities was built. This score ranged between 0 and 55, with higher scores indicating more frequent parent–child interactions. In the present sample, Cronbach’s alpha was 0.88, indicating high internal consistency of the questions.

#### 2.2.3. Behavioral Strengths and Difficulties

Behavioral strengths and difficulties of children were measured using the parent version of the German Strength and Difficulties Questionnaire (SDQ) [29]. The five scales of this instrument assess emotional problems, conduct problems, symptoms of hyperactivity/inattention, peer-relationship problems, and prosocial behavior. The sum scores in each scale range between 0 and 10, with higher scores indicating more behavioral difficulties/strengths. The scores in the problem scales (all scales except prosocial behavior) are summed up to a total behavioral difficulties score. 

Children receiving especially high raw scores in the problem scales or especially low scores in the strength scale (prosocial behavior) can be assigned to “risk” groups of behavioral difficulties. The assignment to a risk group is based on scale-specific cut-off scores obtained in a large normative sample (3 for emotional problems, conduct problems, and peer-relationship problems, 5 for symptoms of hyperactivity/inattention, 12 for total behavioral difficulties, 6 for prosocial behavior) [29]. In the normative sample, 15% of cases received scores above (in the case of prosocial behavior below) these cut-offs and where considered as either “borderline” or “abnormal” [29]. In the present sample, both groups were categorized as “risky”. For further analysis, raw scores as well as the assignment to the “risk” groups were considered.

### 2.3. Data Analysis

All analyses were performed using R version 3.2.2 [30]. “High” screen times were contrasted with “normal” screen times. For children, the categorization in normal versus high was based on current recommendations to limit daily screen times to a maximum of 2 h [31], i.e., all screen times >/= 2 h/day were considered as high. For mothers, we are not aware of any recommendations. Therefore, all screen times higher than the average in the present sample (>/= 5 h/day, see Table 1) were considered as high. All shorter durations were considered as normal. 

The inter-relations between media use of mothers, media use of children, and parent–child interaction were assessed using simple regression analyses. For assessing associations between screen time of mothers, screen time of children, and parent–child interactions (as independent variables) with behavioral strengths and difficulties (as dependent variables), different analyses were performed. In the first analysis, behavioral strengths and difficulties were considered as continuous measures. A more specific analysis focused on the assignments into risk groups of behavioral strengths/difficulties. Further, all associations were investigated using simple regression analyses (including only one independent variable) and multiple regression analyses (including all independent variables simultaneously).

Each association was adjusted for the control variables child age and gender, mother age, and family SES (as continuous measure). Additionally, all statistical models were checked for interactions between the independent variables and age group (2- to 5-year-olds versus 6- to 9-year-olds), child gender, and SES. An interaction between these variables was considered as meaningful if it reached significance (*p* < 0.05) and did not reduce model quality through inflation of variance (variance inflation factor (VIF) <5). Strengths of associations were reported by standardized regression coefficients *β* or by odds ratios OR.

## 3. Results

### 3.1. Media Use of Mothers and Children and Parent-Child Interactions

The average time children and mothers spent watching TV, playing games consoles, using computers and mobile phones, as well as the mean parent–child interactions scores are displayed in Table 1. The statistics are displayed separately for 2- to 5-year-old children (n = 262), 6- to 9-year-old children (n = 291), and the total sample. The medium used most frequently by children was the TV (M = 0.80 h/day). The average total screen time was 1.44 h/day. It was significantly lower for 2- to 5-year-old children (M = 0.91 h/day) than for 6- to 9-year-old children (M = 1.91 h/day) (*p* < 0.001).

For further analysis, all screen times >/= 2 h/day were categorized as “high”. In total, 23% of children (n = 129) were assigned to this group. The remaining ones (77%, n = 424) were assigned to the group of children showing “normal” screen times (<2 h/day). In the age group of 6- to 9-year-olds, “high” screen times were significantly more prevalent (36%) than in the age group of 2- to 5-year-olds (10%) (*p* < 0.001). 

The medium used most frequently by mothers was the mobile phone (M = 1.65 h/day), followed by computers and TV (see Table 1). The average total screen time of mothers was 4.33 h/day. All screen times >/= 5 h/day were categorized as “high” (29%, n = 163). All other screen times were considered as “normal” (71%, n = 390). Neither the total screen time nor the amount of “high” screen times differed significantly between the age groups of 2- to 5-year-old versus 6- to 9-year-old children (all *p* > 0.05). 

The average interactions score was 34.15, indicating that, on average, each of the 11 activities was reported to take place once per week. The shared activities reported most frequently were telling a story/reading, music, moving play, and talking about problems (average response = “>1/week”). Language games and making puzzles were reported the least frequently (average response = “every two weeks”). The interactions score was significantly higher in the group of 2- to 5-year-old children (M = 40.32) than in the group of 6- to 9-year-old children (M = 28.58) (*p* < 0.001). 

### 3.2. Behavioral Strengths and Difficulties of Children

Table 1 displays the average scores on the different scales and the percentage of children assigned to the risk groups of behavioral strengths/difficulties. Again, statistics are presented separately for 2- to 5-year-olds, 6- to 9-year-olds, and the total sample. Regarding behavioral difficulties, peer-relationship problems were reported the least frequently (M = 1.13, percentage of children in risk group = 9%). Symptoms of hyperactivity/inattention were reported most frequently (M = 3.82, children in risk group = 22%). The percentage of children assigned to the risk group of emotional problems differed significantly between 2- to 5-year-olds (10%) and 6- to 9-year-olds (18%) (*p* < 0.01). 

### 3.3. Interrelations between Mothers’ Media Use, Children’s Media Use, and Parent-Child Interaction

High screen times of mothers were significantly associated with high screen times of children (OR = 4.16 (95% CI 2.56–6.76), *p* < 0.001). Children whose mothers reported a high screen time of >/= 5 h/day were more than four times more likely to show high screen time than children whose mothers reported a normal screen time of <5 h/day. In contrast, high screen times of children (β = −0.02 (95% CI −0.10–0.05), *p* = 0.537) and high screen times of mothers (β = −0.004 (95% CI −0.07–0.07), *p* = 0.907) showed no significant association with the amount of reported parent–child interactions. None of the associations differed significantly depending on age group (2- to 5-year-old versus 6- to 9-year-old children), child gender, or SES. 

### 3.4. Associations of Mothers’ and Children’s Media Use and Parent-Child Interaction with Behavioral Strength and Difficulties

**Figure 1:**
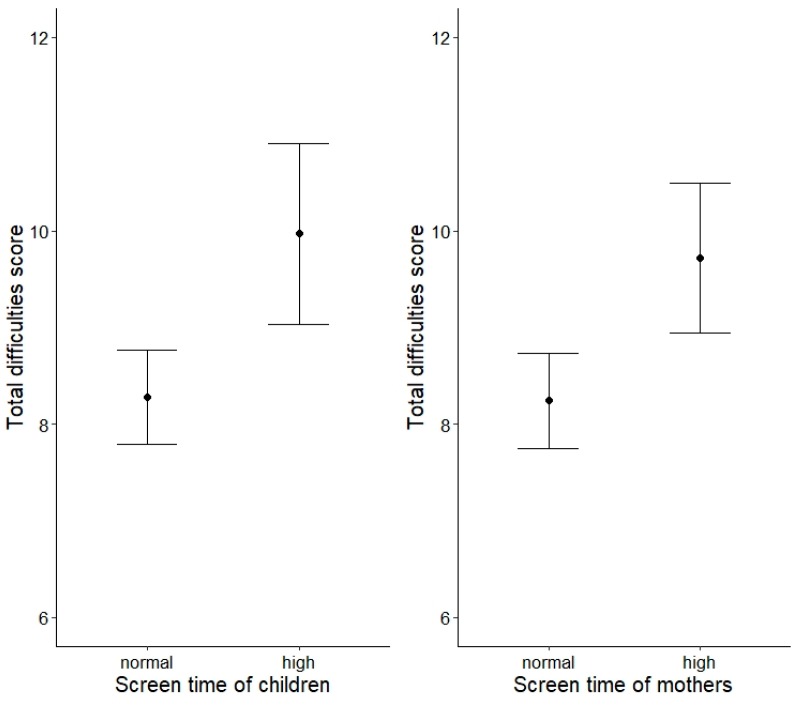
Effect plot (mean effect and 95% CI) illustrating the estimated total behavioral difficulties scores of children depending on screen time of children (on the left) and screen time of mothers (on the right).

**Figure 2:**
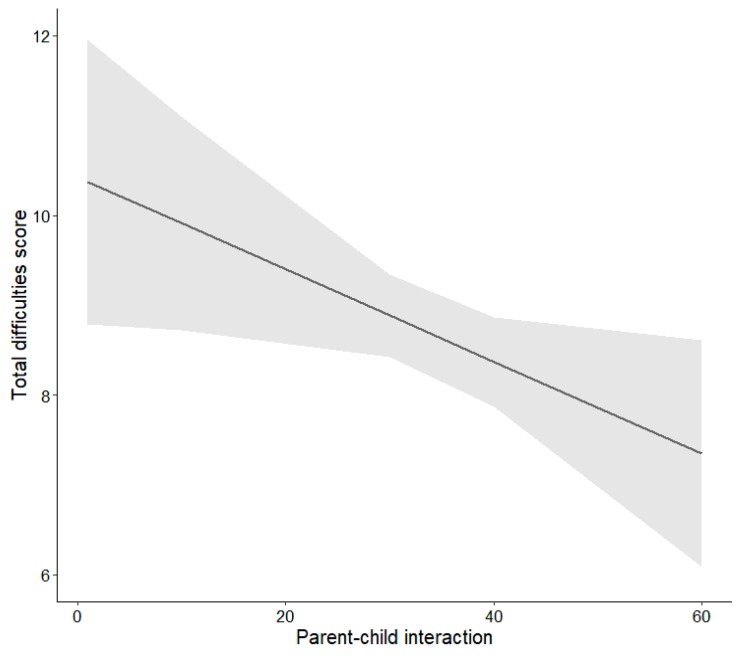
Effect plot (mean effect and 95% CI) illustrating the estimated total behavioral difficulties scores of children depending on parent–child interaction scores.

High screen time of children (β = 0.14, *p* = 0.003), high screen time of mothers (β = 0.13, *p* = 0.002), and lower parent–child interaction scores (β = −0.12, *p* = 0.029) were significantly associated with higher total behavioral difficulties scores of children. As can be seen in Figure 1, the total difficulties score was estimated to be 1.69 points higher in children showing high screen times (>/= 2 h/day) than in children showing normal screen times (<2 h/day). The estimated difference between children of mothers showing high versus normal screen times was 1.48 points (see Figure 1). The association between parent–child interactions and the total difficulties score is displayed in Figure 2. In a model in which media use of children, media use of mothers, and parent–child interaction were included simultaneously, all associations remained significant (all *p* < 0.05), indicating that the associations were independent.

Table 2 displays the associations of media use and parent–child interaction with the scores on the single scales of the SDQ. As can be seen, a high screen time of children was associated with more conduct problems (β = 0.12, *p* = 0.013), more symptoms of hyperactivity/inattention (β = 0.13, *p* = 0.005), and less prosocial behavior (β = −0.12, *p* = 0.007). A high screen time of mothers was associated with more emotional problems (β = 0.10, *p* = 0.014), more conduct problems (β = 0.11, *p* = 0.013), and more symptoms of hyperactivity/inattention (β = 0.09, *p* = 0.039) in children. Higher parent–child interaction scores were significantly associated with fewer conduct problems (β = −0.13, *p* = 0.013), fewer peer-relationship problems (β = −0.14, *p* = 0.007), and more prosocial behavior (β = 0.25, *p* < 0.001) in children. If media use of children, media use of mothers, and parent–child interaction were included as independent variables in the same models (multiple regression), the associations of screen time of children with hyperactivity/inattention and prosocial behavior, the relation between screen time of mothers and emotional problems, and the associations of parent–child interactions with conduct problems, peer-relationship problems, and prosocial behavior remained significant (all *p* < 0.05). None of the associations differed significantly depending on age group (2- to 5-year-old versus 6- to 9-year-old children), child gender, or SES.

In a more specific analysis, we investigated if media use of children, media use of mothers, and parent–child interactions related to especially high behavioral difficulties scores or especially low behavioral strengths scores (assignment to a “risk” group). The analyses showed that a high screen time of mothers was significantly related to a higher probability of belonging to the risk group of emotional problems (OR = 1.79 (95% CI 1.06–3.00), *p* = 0.028). Higher amounts of parent–child interactions were significantly associated with a lower probability of belonging to the risk groups of conduct problems (OR = 0.97 (95% CI 0.95–0.99), *p* = 0.029), and prosocial behavior (OR = 0.94 (95% CI 0.92–0.98), *p* = 0.001), and with a lower risk of high total behavioral difficulties scores (OR = 0.98 (95% CI 0.96–0.99), *p* = 0.045). All other associations did not reach significance (all *p* > 0.05). 

## 4. Discussion

This study explored associations between facets of parent–child behavior (screen time of children, screen time of mothers, frequency of parent–child interactions) and behavioral strengths and difficulties in 2- to 9-year-old children. In the present sample, 2- to 5-year-old children spent approximately 1 h per day using different screen-based media. The average daily screen time of 6- to 9-year-old children was nearly 2 h, which is comparable to (though slightly lower than) the screen times reported in a national German survey (KIM study) on the media use of 6- to 13-year-old children [1]. Importantly, more than 20% of children exceeded current recommendations to limit daily screen time to a maximum of 2 h [31]. This finding demonstrates the (potentially detrimental) significance of electronic media in the lives of children and the importance to strengthen the media competence of children and their parents. The average daily screen time of mothers was approximately 4 h. This is comparable to the screen times reported by parents participating in the Kim study [1]. 

With respect to parent–child interactions, the present data show that, on average, all activities assessed in this study were reported to take place at least every 2 weeks but not more frequently than several times per week. The data, furthermore, showed that parents interacted more frequently with younger than with older children. In contrast to younger children, older children might be more independent and interact more frequently with peers than with parents. Furthermore, at school age, parent–child interactions might include more academic activities, which were underrepresented in the questionnaire applied here. 

Overall, the average scores on the different scales of the SDQ were comparable to the average scores reported in the KiGGS study, a large nationwide survey on the health of children growing up in Germany [32]. 

### 4.1. Interrelations between Mothers’ Media Use, Children’s Media Use, and Parent–Child Interaction

As hypothesized, screen time of children was significantly associated with screen time of mothers. This is in line with the findings of several other studies [18,19]. A possible reason for this association is that parents and children use electronic media together, e.g., watch TV together or play a mobile game together [33]. Another reason might be that children use their mothers as role models. Seeing others using mobile phones or watching TV might create the desire to use these devices by oneself. Finally, the shared media environment at home might impact both media use of mothers and children and, therefore, trigger an association between both.

In contrast to our expectations and previous studies [8,20], media use of mothers and media use of children were not significantly related to the amount of parent–child interactions. This finding suggests that the time mothers or children spent in front of a screen does not displace shared parent–child interactions. However, due to the study design applied here, we are not able to tell how frequently media were used in times spent with the family. Furthermore, we did not assess the quality or the duration of shared activities and the person (mother versus father) the activity was shared with. Future studies might explicitly assess media use during parent–child interactions and investigate associations with quality and duration of interactions. 

### 4.2. Associations of Mothers’ And Children’s Media Use and Parent–Child Interactions with Behavioral Strengths and Difficulties

The findings of the present study largely confirmed the hypotheses that media use of children and parents is associated with more behavioral difficulties and less prosocial behavior, whereas parent–child interaction is related to fewer behavioral difficulties and more prosocial behavior. 

A high screen time of children and mothers and a lower frequency of parent–child interactions were independently associated with more behavioral difficulties in children. As already shown in other cross-sectional studies [8,9], excessive media use of children was related to more externalizing problems, namely conduct problems (e.g., anger, disobedience) and symptoms of hyperactivity/inattention (low attention span, restlessness). The associations with hyperactivity/inattention and prosocial behavior remained significant after controlling for media use of mothers and parent–child interactions. In young children, a high media use is argued to cause an overstimulation of the brain, to distract children, and to go along with a displacement of other activities such as physical or social activities [8,34]. These factors might, in turn, have a negative impact on attention span, frustration tolerance, and social competencies [8]. Regarding the association between media use and symptoms of hyperactivity/inattention, findings of longitudinal studies in this field strengthen this interpretation [4,10]. However, the causal relationships between media use and signs of hyperactivity or inattention and underlying mechanisms are still not well understood [35]. For example, children suffering from ADHD have been shown to be at special risk for developing gaming disorders [36,37], indicating that behavioral problems might also cause an overuse of electronic media. It is, furthermore, possible that parents of children showing more externalizing and antisocial behavior authorize a higher media use, e.g., in order to calm or to occupy them [38]. 

With respect to screen time of mothers, we observed associations with externalizing problem behavior (conduct problems, symptoms of hyperactivity/inattention) but also with internalizing problem behavior (emotional problems, e.g., sadness, depressive mood). These findings are in line with a study in which media use of mothers was associated with interruptions of parent-child interactions, which, in turn, were related to more externalizing and internalizing problem behavior of 1- to 5-year-old children [20]. However, in the present study, only the association with emotional problems remained significant after controlling for media use of children and parent–child interactions. Importantly, high media use of mothers was also associated with a higher probability of belonging to a risk group of emotional problems, i.e., with especially high scores in this problem scale. A possible reason for the associations between media use of mothers and behavioral difficulties of children is that electronic media use reduces mothers’ attention and responsiveness, especially if media are used in the presence of the child [13,14,15,20]. This might frustrate children, and emotional responses and disobedience might represent two ways to express this frustration [15]. On the other hand, mothers whose children suffer from behavioral problems might seek for social support and information on the internet and social media or use electronic media to get distracted and to escape the frustrations of parenting [39]. It is, furthermore, possible that other personal factors such as emotion regulation problems of mothers [40] influence both maternal electronic media use and child behavior. 

Concerning parent–child interactions, the strongest associations were observed with conduct problems, peer-relationship problems (e.g., problems with peers, social exclusion), and prosocial behavior, independently of the media use of children and mothers. These findings are in line with previous studies [8,22]. Importantly, lower amounts of parent–child interactions were also significantly associated with the probability of belonging to the risk groups of conduct problems or prosocial behavior, i.e., of showing relevant amounts of antisocial behavior. Similar to high media use of mothers, infrequent parent–child interaction might frustrate children, and disobedience, a lack of respect and helpfulness, and social problems might reflect a way of coping with this frustration. Furthermore, peer problems and social withdrawal might be a consequence of a lack of social competences [41]. At the same time, children with social or conduct problems might be less interested in parent–child interactions and have more difficulties to engage in shared activities.

### 4.3. Limitations

The SES of study participants was very high and, therefore, not representative for German families. A further fact that should be considered when interpreting the data is that the categorization of screen times to “normal” or “high” was based on current recommendations and observations made in this study. Therefore, not all screen times categorized as “normal” might necessarily be healthy (especially in the younger age group). Also, the media questionnaire did not explicitly distinguish between media usage for entertainment versus academic purposes. 

An additional limitation of the study is that all measures were based on reports of mothers. Due to the strong under-representation of fathers in the LIFE Child study, their perspectives and behaviors could not be considered. Most importantly, it was not assessed if mothers used media during mother–child interactions or only in the absence of the child. In the context of parent–child interactions and child behavior, information on media use during interaction with the child might be much more essential than information on media use in general. Finally, only cross-sectional data were reported in this study. Therefore, assumptions on possible causal relationships remain speculative. Future studies might apply a longitudinal design and consider screen time and interactive behavior of mothers, fathers, and other family members living in the same household. Additionally, measurements should explicitly focus on parental media use in the presence of the child. Regarding the media use of children at home, different purposes of usage (e.g., for entertainment or homework) might be distinguished.

## 5. Conclusions

The present findings suggest that the media use of children is associated with the media use of their mothers and that both high screen times of children and of mothers are more frequent in children showing behavioral difficulties. At the same time, the study showed that frequent parent–child interaction is associated with fewer behavioral difficulties, independently of media use within the family. These findings underline the importance of shared activities and the potentially detrimental role of electronic media use in the family context. They, furthermore, indicate that parents should be required to keep in check both the media use of their children and their own behavior.

## Figures and Tables

**Table 1:** Means (and standard deviations) of screen times, parent–child interaction scores, and SDQ scores in the present sample.

	Children				Mothers
Measure	Possible Range	2–5 years n = 262	6–9 years n = 291	All n = 553	All n = 553
**Media use**					
TV	0–5	0.63 (0.51)	0.96 (0.69)	0.80 (0.63)	1.23 (1.01)
Games console	0–5	0.01 (0.08)	0.15 (0.14)	0.09 (0.26)	0.03 (0.15)
Computer	0–10	0.18 (0.35)	0.49 (0.85)	0.34 (0.68)	1.42 (1.48)
Mobile phone	0–10	0.09 (0.20)	0.31 (0.83)	0.21 (0.62)	1.65 (1.52)
Total screen time ^a^	0–30	0.91 (0.71)	1.91 (2.00)	1.44 (1.61)	4.33 (2.91)
n (%) high screen time ^b^		26 (10%)	103 (36%)	129 (23%)	163 (29%)
**Parent–child interactions**					
Score	0–55	40.32 (7.43)	28.58 (11.40)	34.15 (11.35)	
**Behavioral strengths and difficulties**					
Emotional problems	0–10	1.51 (1.63)	1.81 (1.86)	1.67 (1.76)	
n (%) risk group		26 (10%)	52 (18%)	78 (14%)	
Conduct problems	0–10	2.26 (1.54)	1.89 (1.61)	2.07 (1.59)	
n (%) risk group		52 (20%)	41 (14%)	93 (17%)	
Hyperactivity inattention	0–10	3.94 (2.31)	3.72 (2.59)	3.82 (2.46)	
n (%) risk group		55 (21%)	69 (24%)	124 (22%)	
Peer-relationship problems	0–10	1.16 (1.42)	1.09 (1.53)	1.13 (1.48)	
n (%) risk group		21 (8%)	28 (10%)	49 (9%)	
Total behavioral difficulties	0–40	8.87 (4.55)	8.50 (5.50)	8.68 (5.07)	
n (%) risk group		55 (21%)	63 (22%)	118 (21%)	
Prosocial behavior	0–10	7.60 (1.67)	8.07 (1.75)	7.85 (1.72)	
n (%) risk group		30 (11%)	23 (8%)	53 (10%)	

^a^ Total screen time = combination of TV, games console, computer, and mobile phone use. ^b^ High screen time = >/= 2 h/day (for children) and >/= 5 h/day (for mothers). SDQ = Strength and Difficulties Questionnaire.

**Table 2:** Associations of mothers’ and children’s media use and parent–child interactions with behavioral strengths and difficulties (n = 553 2- to 9-year-old children and their mothers).

	Dependent Variable: Behavioral Strengths and Difficulties of Children				
Independent Variables	Emotional Problems	Conduct Problems	Hyperactivity/Inattention	Peer-Relationship Problems	Prosocial Behavior
	β (95% CI)	β (95% CI)	β (95% CI)	β (95% CI)	β (95% CI)
Screen time children ^a^	0.06 (−0.03–0.15)	0.12 (0.02–0.21) *	0.13 (0.04–0.22) **	0.07 (−0.02–0.16)	−0.12 (−0.21 to −0.03) **
Screen time Mothers ^a^	0.10 (0.02–0.19) *	0.11 (0.02–0.19) *	0.09 (0.01–0.17) *	0.07 (−0.01–0.16)	0.04 (−0.03–0.13)
Parent–child interactions	0.01 (−0.10–0.11)	−0.13 (−0.23 to−0.03) *	−0.07 (−0.17–0.03)	−0.14 (−0.25 to −0.04) **	0.25 (0.15–0.35) ***

All associations are adjusted for child age, child gender, mother age, and family SES. ^a^ Reference = normal screen time (<2 h/day for children, <5 h/day for mothers); * *p* < 0.05, ** *p* < 0.01, *** *p* < 0.001; *β* = standardized regression coefficient.
